# Pupil diameter changes reflect difficulty and diagnostic accuracy during medical image interpretation

**DOI:** 10.1186/s12911-016-0322-3

**Published:** 2016-07-05

**Authors:** Tad T. Brunyé, Marianna D. Eddy, Ezgi Mercan, Kimberly H. Allison, Donald L. Weaver, Joann G. Elmore

**Affiliations:** Center for Applied Brain and Cognitive Sciences, 200 Boston Ave, Suite 3000, Medford, 02155 MA USA; Department of Psychology, Tufts University, 490 Boston Ave, Medford, 02155 MA USA; Department of Computer Science and Engineering, University of Washington, Seattle, 98104 WA USA; Department of Pathology, Stanford University School of Medicine, Palo Alto, 94305 CA USA; Department of Pathology and UVM Cancer Center, University of Vermont, Burlington, 05401 VT USA; Department of Medicine, University of Washington, Seattle, 98104 WA USA

**Keywords:** Decision making, Medical image interpretation, Pupil diameter, Locus coeruleus, Diagnosis

## Abstract

**Background:**

No automated methods exist to objectively monitor and evaluate the diagnostic process while physicians review computerized medical images. The present study tested whether using eye tracking to monitor tonic and phasic pupil dynamics may prove valuable in tracking interpretive difficulty and predicting diagnostic accuracy.

**Methods:**

Pathologists interpreted digitized breast biopsies varying in diagnosis and rated difficulty, while pupil diameter was monitored. Tonic diameter was recorded during the entire duration of interpretation, and phasic diameter was examined when the eyes fixated on a pre-determined diagnostic region during inspection.

**Results:**

Tonic pupil diameter was higher with increasing rated difficulty levels of cases. Phasic diameter was interactively influenced by case difficulty and the eventual agreement with consensus diagnosis. More difficult cases produced increases in pupil diameter, but only when the pathologists’ diagnoses were ultimately correct. All results were robust after adjusting for the potential impact of screen brightness on pupil diameter.

**Conclusions:**

Results contribute new understandings of the diagnostic process, theoretical positions regarding locus coeruleus-norepinephrine system function, and suggest novel approaches to monitoring, evaluating, and guiding medical image interpretation.

## Background

Interpreting medical images involves dynamic interactions between the perception of visual features and the application of knowledge. This visual interpretive process is fundamental to arriving at accurate diagnoses across several medical specialties including pathology, radiology, and dermatology. However, interpreting medical information and arriving at a diagnosis is a highly subjective processes and little is known about how to measure, predict, and optimize it in education and clinical practice. More detailed scientific understandings of the applied medical interpretive process will contribute to theory development and begin identifying objective, quantitative metrics for training and assessing competency. Medical educators will benefit from reliable and objective methods for providing feedback, assessing competence, and advancing student learning.

Within the domain of pathology, the advent of digital whole slide imaging has made it possible to monitor eye movements in real-time while pathologists interpret the images of biopsied tissue from glass slides shown on a computer screen. Research on whole slide images has demonstrated that novice and expert pathologists move their eyes differently, focus on different elements of the visual image, and often arrive at very different diagnostic decisions [1–3]. These new metrics make it possible to objectively assess student progress in innovative ways without directly probing subjective knowledge. The present study extends this research to examine whether pupil dilation during visual image interpretation might reveal reliable differences based on case difficulty, diagnostic category of the biopsy findings, or the convergence with consensus reference diagnoses. This research is motivated by current theories of locus coeruleus-norepinephrine system function, which suggest that pupillometry may be a useful tool for monitoring and characterizing ongoing cognitive states [4–6].

In general, pupil diameter is modulated by both environmental (e.g., lighting conditions) and psychological processes, and is considered a reliable measure of mental engagement. Recent empirical work has identified ongoing (tonic) and event-related (phasic) variations in pupil diameter as valuable for assessing a range of perceptual and attentional phenomena [5]. Increased tonic pupil diameters throughout the duration of a task have been shown to correlate with increased mental effort toward resolving visual features and solving a task. For instance, larger pupil diameters are found when visual stimuli are difficult or ambiguous to discriminate, and problem sets are computationally demanding [7, 8]. Tonic variation is believed to reflect the diffuse deployment of attention to enable exploration of a visual stimulus [7]. In contrast to tonic variations, event-related phasic changes in pupil diameter relate to a range of acute task demands. For instance, the pupil quickly and reliably dilates when perceived visual information is highly relevant to solving a search-related task [9]. Phasic variation is believed to reflect focused attention when exploiting current information and disregarding extraneous details [10], though this phenomenon has not been investigated within the domain of diagnostic interpretation.

Tonic and phasic variation of pupil diameter are thought to reflect modulation of the locus coeruleus-norepinephrine (LC-NE) system. The LC is located in the dorsal pons and exerts influence via NE projections throughout the forebrain; though the precise neuromodulatory relationship between LC-NE function and pupil diameter is unknown, empirical evidence demonstrates reliable positive relationships between LC neuronal firing rates and increases in pupil diameter [11, 12]. Accounting for these data, one recent theory suggests that modulations in pupil diameter reliably index cognitive control states and indirectly indicate LC-NE system function [4]. In particular, the adaptive gain theory suggests that the LC-NE system regulates the balance between exploitation and exploration [4, 5]; exploitation involves continuing to pursue a potential reward source, and exploration involves searching for new reward sources. While interpreting medical images, pathologists likely exploit particular areas (high salience, high diagnostic value [3]) of a visual scene toward forming a diagnostic interpretation, and also explore the visual scene to scan for additional regions of potential diagnostic value. Interestingly, some current models of pupil response also suggest that phasic dilation can occur prior to, and perhaps is even necessary for, awareness of viewing relevant visual information [5, 7]. In other words, event-related pupil dilation is thought to not only occur when a searched for target is fixated on but may also be necessary for a person to become aware of seeing the target and assessing its relevance. Overall, pupil diameter appears to reliably reflect the ongoing tonic demands of a task, and further shows phasic sensitivity to the task relevance of encountered visual stimuli; these findings allow us to generate testable predictions in the domain of diagnostic pathology.

Several case- and physician-level factors may modulate tonic and phasic pupil diameter during interpretation, though very few studies have specifically examined dynamic changes in pupil diameter during the medical diagnostic process. In one, pupil diameter was measured while anesthetists participated in simulated anesthesia induction; the authors found that pupil diameter increased as the severity of simulated critical conditions increased [13]. Similar results have been found with surgeons in simulated operating rooms [14]. Some recent research suggests that pupil responses depend on task demands and the experience level of physicians, and may predict ultimate diagnostic accuracy [15–18]. None of these studies, however, examined tonic and phasic pupil responses on behalf of pathologists interpreting whole slide images. Based on this earlier work and broader findings in the perceptual and cognitive sciences literature, we can make several hypotheses.

Given that more difficult visual stimuli tend to elicit higher tonic pupil diameters, we expect that ratings of case difficulty will modulate tonic pupil diameter throughout interpretation. Cases that tend to elicit higher difficulty ratings (and diagnostic discordance among pathologists) [19] should be associated with higher pupil diameters. This result would reflect engagement of exploratory search processes toward identifying diagnostically relevant image regions. We expect this pattern to emerge with both resident and faculty pathologists.

We expect pupil diameter to show phasic changes when pathologists fixate in a pre-defined diagnostically relevant region. Given that phasic pupil diameter is quickly guided by the task relevance of stimuli [7], if a diagnostic region is deemed task relevant by a pathologist then we expect them to engage exploitation processes. Engaging exploitation processes should increase pupil diameter upon fixation in task-relevant diagnostic regions. Thus, we expect higher pupil diameters when pathologists’ fixate in a pre-defined diagnostically relevant region of interest. We also expect this pattern to be modulated by case difficulty and whether a pathologist’s ultimate diagnosis agrees with consensus reference. The former hypothesis suggests that pupil responses may be more pronounced with difficult cases because identifying a region of diagnostic importance is particularly relevant when a case involves greater effort toward resolution. Though exploratory, the latter hypothesis suggests that phasic changes in pupil diameter may predict diagnostic agreement. We aim to test both of these hypotheses by using an eye tracking system to monitor pupil diameter while pathologists interpret and diagnose digitized breast biopsies.

## Methods

### Participants

We recruited and collected data from 22 physicians from major U.S. university medical centers, one on the east coast (*n* = 11) and one on the west coast (*n* = 11). Physicians had a range of experience interpreting breast pathology: 6 faculty members specializing in breast and general anatomic pathology, and 16 residents with limited breast pathology experience. Recruitment and data collection were completed from May 2014 to January 2015. Participants provided written informed consent. All materials and study activities had primary Institutional Review Board approval from the Fred Hutchinson Cancer Research Center, including approval to physically travel with an eye-tracking device to off-site data collection sites. Note that no approvals were pursued from the west coast data collection site’s IRB because participants enrolled at that site were not associated with the grant or engaged in research. Rather, they enrolled in the protocol as private individuals, not as employees of the university medical center.

### Materials & equipment

A test set of 24 hematoxylin and eosin stained digital whole slide image breast specimens was selected from a larger (240 case) test set used as part of an ongoing National Cancer Institute (NCI) funded breast pathology study [19, 20]. Cases were gathered from women ranging in age from 40 to 50+ years, with varying breast density. Using a method described elsewhere [21] each specimen was associated with a single reference diagnosis based on consensus of three experienced breast pathologists. These experts also identified at least one diagnostic region of interest (dROI) per specimen that they deemed the “best example (s)” of the consensus reference diagnosis. The 24 cases spanned four diagnostic categories, with greater representation of cases associated with diagnostic discordance according to data [19] from 115 practicing pathologists: four benign without atypia cases, eight atypia, eight ductal carcinoma in situ (DCIS), and four invasive cancer. Within each diagnostic category, half of the cases were rated as low and half relatively high difficulty based on subjective ratings (scale 1–6, with 1 indicating lowest difficulty, and 6 indicating highest difficulty) data gathered from a large sample of pathologists (*N* = 115; ${\overline{x}}_{\mathrm{low}}$ = 2.26; ${\overline{x}}_{\mathrm{high}}$ = 3.27). We did not select any cases that consistently elicit high difficulty ratings (indicating potential discordance); selected cases had a minimum rating of 1.31 and maximum of 4.03 (scale 1–6). Glass slides were scanned into digital TIFF format using an iScan Coreo Au digital slide scanner [22] at 40× magnification. The test set was divided into two subsets of 12 cases (2 benign, 4 atypia, 4 DCIS, and 2 invasive), each with similar ratings across the two difficulty levels. We chose to use 12 cases to ensure that pathologists could reasonably interpret all cases within a 1-h experiment session.

We used the non-invasive RED-m portable eye tracking system manufactured by SensoMotoric Instruments (SMI; Boston, MA). The infrared camera-based system tracks gaze position and pupil data at 60Hz with high angular accuracy (0.5°) and spatial resolution (0.1°). The system uses a 9-point calibration process to achieve angular accuracy. The system was mounted to the bottom of a 22” flat screen LCD monitor running at 1920 × 1080 resolution, and participants were seated at a distance of 60 cm from the monitor.

A digital slide viewer was developed to display images in a navigable viewport that allows zooming (1–60×) and panning while maintaining full image resolution. The viewport automatically logged participant behavior over time, including current view position and zoom level. Participants indicated their final diagnosis on each case by selecting from four diagnostic categories (benign without atypia, atypia, DCIS, and invasive).

### Data collection locations & procedures

At each data collection location participants were seated in a private conference room with the experimenter. Following consent, participants completed eye tracker calibration, involving watching a dot move between nine points on the screen. Participants were then instructed how to interact with the image viewer. Each participant then viewed one of the two subsets of 12 cases, at full screen, in random order. After each case they indicated their final diagnosis and rated case difficulty on a scale from 1 to 6. To reduce possible decision biases, participants were instructed to interpret slides as they would in clinical practice. All participants were remunerated with a $50USD gift card.

### Statistical analysis

To assess diagnosis accuracy, pathologists’ interpretations were compared to the consensus reference diagnosis. We entered the agreement rates into a mixed analysis of variance (ANOVA) with diagnostic category as the within-participants’ factor and expertise as the between-participants’ factor.

The eye tracking system outputs the Cartesian coordinates of the eye, and pupil diameter (in pixels), over time. In the event of eye blinks, we used linear interpolation to rectify null samples. To assess tonic pupil diameter we used raw pupil diameter data over time, using the fast Fourier transform method (MATLAB “interpft” command) to normalize case interpretation time across all cases and participants. We then divided normalized time into 10 time increments covering the pathologists’ interpretation of cases (samples 1–50, 50–100, etc.).

To assess whether tonic pupil diameter varied as a function of case difficulty, we parsed cases based on ratings provided by the present participant sample. In the event of missing data for particular difficulty levels we replaced with condition means. Because the 6-point rating scale produced relatively sparse ratings data from the tails of the rating scale (i.e., 1 and 6; resulting in highly unequal data points per condition), we reduced data to 4 conditions (A: ratings 1 and 2; B: rating 3; C: rating 4; D: ratings 5 and 6). This resulted in a similar number of trials in each of the 4 conditions (66, 57, 48, and 43, respectively). Tonic pupil data were analyzed using a repeated-measures ANOVA with two within-participants factors: difficulty (4 levels) and time (10 increments). Note that baseline pupil diameter during the first 60 samples (1 s) of case interpretation did not vary as a function of difficulty, *F*(3, 60) = 1.75, *p* = .17; thus, raw (rather than relative) data are used for analyses of tonic pupil diameter.

To assess phasic pupil diameter, we converted the raw coordinate data into fixations and saccades using conventional methods [23] and then identified the point in time when the eye first fixated in one of the regions of interest on each case. This point in time was used as a reference for extracting baseline pupil diameter 100 milliseconds prior to (6 samples) and 5 s following (300 samples) the first fixation in a consensus dROI. We chose 5 s given earlier work demonstrating that this time window can capture both the phasic response and subsequent return toward baseline pupil diameters [24]. Baseline samples preceding the first dROI fixation were averaged and then all 300 subsequent samples were referenced to this baseline to evaluate relative pupil diameter upon arrival in dROI. To analyze phasic pupil diameter we conducted a repeated-measures ANOVA with two factors: diagnosis agreement with consensus (true, false) and participant-rated case difficulty (low, high). Diagnosis agreement was operationalized as convergence with consensus reference diagnosis; case difficulty groups were developed via median split on rated (by the present sample) difficulty. Categorizing cases in this 2 × 2 matrix allowed us to isolate the following case types:

- Cases pathologists considered relatively easy but ultimately failed to arrive at a diagnosis that agreed with the consensus reference diagnosis.
- Cases pathologists considered relatively difficult and ultimately failed to arrive at a diagnosis that agreed with the consensus reference diagnosis.
- Cases pathologists considered relatively easy and ultimately arrived at a diagnosis that agreed with the consensus reference diagnosis.
- Cases pathologists considered relatively difficult and ultimately arrived at a diagnosis that agreed with the consensus reference diagnosis.

Because changes in screen brightness can modulate pupil diameter, we used video screen captures to extract frame-by-frame mean grayscale intensity values with MATLAB (Mathworks, Inc., Natick, MA). To adjust pupil diameter due to screen brightness, we divided pupil diameter by the recriprocal of this intensity value; in this manner, lower image intensity values would potentially account for pupil dilation over time. To ensure any results were not driven by screen brightness alone, tonic and phasic pupil diameter analyses were rerun using these adjusted values.

## Results

One participant’s data, and 38 of the 252 remaining cases, were removed due to exceedingly poor eye tracking quality. The remaining data (214 cases; 21 participants) were used for analyzing pathologists’ tonic pupil diameter, and diagnostic agreement with consensus.

### Agreement with expert consensus reference diagnosis

Agreement varied as a function of the four diagnostic categories, *F*(3, 60) = 21.16, *p* < .001, and by physicians’ expertise, *F*(1, 19) = 5.78, *p* < .05, with no interaction between these two (*p* = .97). For the effect of diagnostic category, there was highest agreement in the invasive cancer (*M* = .91), moderate agreement in the benign without atypia cases (*M* = .71), and lowest agreement in the atypia and DCIS conditions (*M* = .39, and *M* = .35, respectively), matching the overall pattern found in recent research [19]. Follow-up paired t-tests demonstrated differences (*p’s* < .05) between all pairwise comparisons other than atypia versus DCIS (*p* = .53). For the effect of expertise, there was higher agreement in the faculty group (*M* = .70) relative to the resident group (*M* = .55).

### Tonic pupil diameter

A repeated-measures ANOVA with 4 difficulty levels and 10 time increments revealed a main effect of difficulty, *F*(3, 60) = 5.62, *p* < .01. Overall, larger pupil diameters were found in the higher rated difficulty cases (see Table 1), supporting our hypothesis.

**Table 1:** Mean absolute tonic pupil diameter (in pixels) for the four difficulty conditions (derived from participant ratings) and ten time bins

	Time 1	Time 2	Time 3	Time 4	Time 5	Time 6	Time 7	Time 8	Time 9	Time 10	Overall mean
Least difficult	13.41	13.32	13.33	13.27	13.32	13.37	13.29	13.39	13.36	13.26	13.33
Somewhat difficult	13.47	13.46	13.50	13.56	13.51	13.46	13.65	13.38	13.40	13.30	13.47
Moderately difficult	13.61	13.59	13.62	13.65	13.61	13.60	13.69	13.69	13.69	13.69	13.64
Most difficult	14.03	13.93	13.95	14.03	13.93	13.90	13.87	13.92	13.89	13.91	13.94

None of these patterns was modulated by participant expertise (resident, faculty), reference diagnostic category, or whether the participant diagnosis converged with the consensus reference diagnosis. To ensure our data were not due to image intensity differences, we reran the 4 × 10 ANOVA with adjusted pupil diameter values (see Method section); the ANOVA still showed the main effect of difficulty, *F*(3, 60) = 4.91, *p* < .01.

### Phasic pupil diameter

**Fig. 1:**
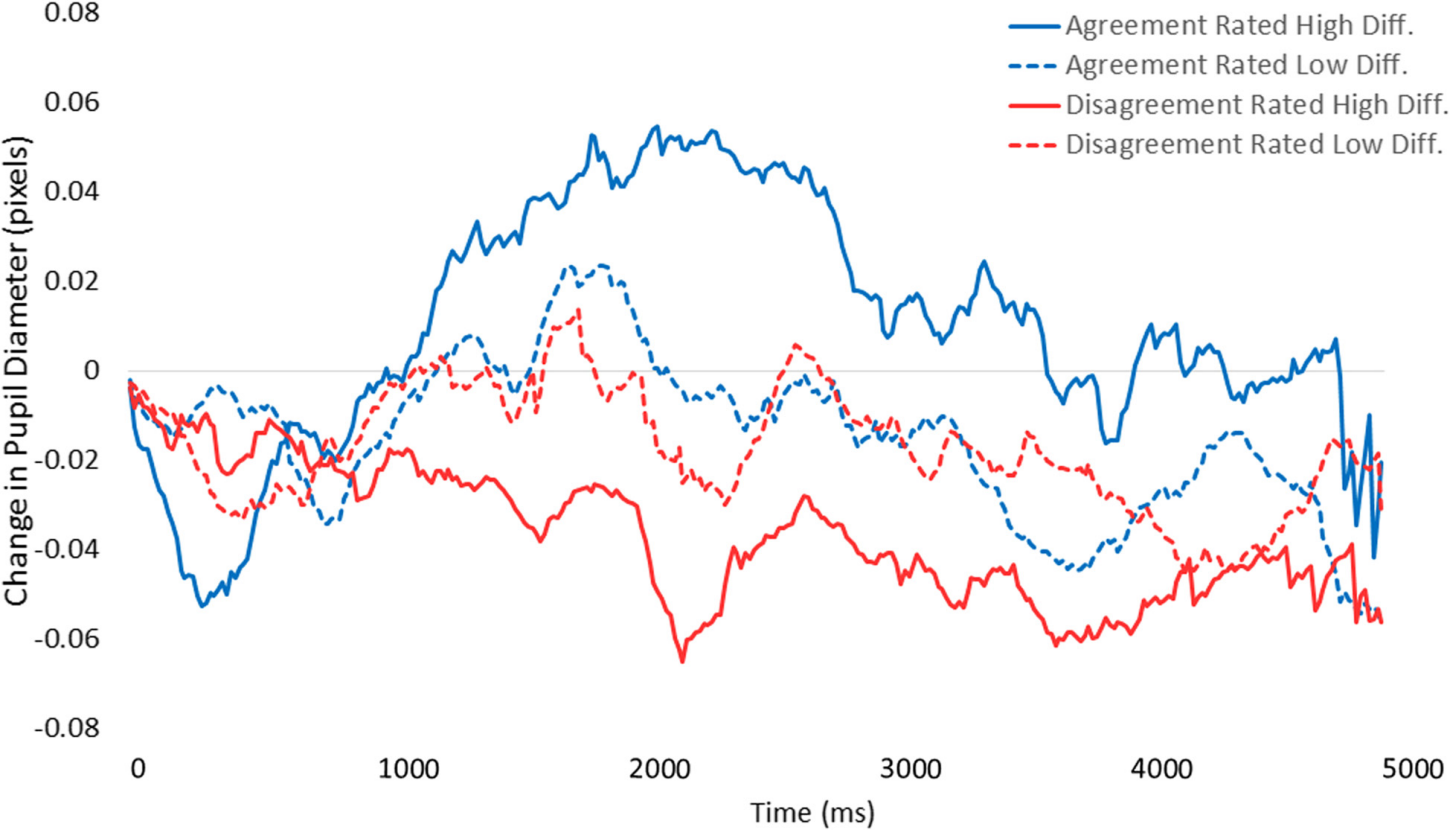
Mean change in pathologists’ pupil diameter relative to baseline (in pixels) as a function of rated case difficulty and whether their diagnosis agreed or disagreed with the consensus reference diagnosis

Participants fixated in a dROI on 205 cases (81.3 % of cases); four of these cases were removed due to greater than 50 % null samples surrounding the reference time point. This resulted in 201 trials (61,506 pupil diameter samples) remaining for analysis of phasic response. Raw data are depicted in Fig. 1. For the ANOVA, we parsed time into 10 bins each containing 500 milliseconds (30 samples) of data. The ANOVA revealed a main effect of time, *F*(9, 180) = 3.04, *p* < .01, and no main effect of trial type, *F*(3, 60) = .78, *p* > .05. The two factors interacted, *F*(27, 540) = 1.88, *p* < .01. As depicted in Fig. 1, when cases were rated difficult but participants ultimately agreed with the consensus reference diagnosis, pupil diameter showed the largest increase. In contrast, when cases were rated low difficulty and participants disagreed with the consensus reference diagnosis, pupil diameter showed a decrease.

Overall, it appears that cases rated low in difficulty elicited very little modulation of pupil diameter, regardless of whether the diagnosis agreed with consensus. More difficult cases, however, elicited either increased diameter or decreased diameter based upon whether participants ultimately arrived at, or did not arrive at, agreement with the consensus reference diagnosis on the case (respectively).

This pattern maintained for both residents and faculty, and did not reliably differ as a function of consensus reference diagnostic category of the case. As with tonic diameter, we reran the phasic analyses after accounting for mean image intensity over time; the interaction between time and trial type persisted, *F*(27, 540) = 1.61, *p* < .01.

## Discussion and conclusions

The present pilot study was designed to test for tonic and phasic modulation of pupil diameter while pathologists interpreted and diagnosed digitized whole slide images of breast biopsies. In accordance with recent theories of the LC-NE system’s role in modulating pupil diameter through dynamic changes in exploitation-exploration cognitive processes, we made two primary hypotheses. First, we expected that tonic pupil diameter would be influenced by perceived difficulty of a case, reflecting the overall engagement of exploratory search processes. Second, we expected that phasic pupil diameter upon fixating in a predefined diagnostic region of interest would be modulated by case difficulty, and predict whether a diagnosis converged with consensus reference diagnosis. This pattern would demonstrate the recognition of task-relevant visual features and engagement of exploitation-based processes. Overall, we found support for these hypotheses, and also identified some patterns that motivate continuing research.

Tonic pupil diameter has been shown to reliably correlate with the level of mental effort involved in processing and interpreting visual information [7]. Data from this study support and extend this concept among physicians processing and interpreting visual medical data, demonstrating that biopsy cases associated with higher levels of perceived clinical difficulty elicited the largest pupil diameters. Using individual pathologists’ difficulty ratings, we found that the lowest tonic pupil diameters were noted in the low difficulty cases, and there was a step-wise increase in pupil diameter corresponding to the pathologists’ difficult ratings of biopsy cases. Thus, tonic pupil diameter may be a useful, unintrusive indicator of perceived difficulty during physicians’ interpretive process. Pupil diameter data may prove a reliable surrogate for subjective difficulty ratings in training contexts, objectively assessing the mental effort a pathologist is exerting toward interpreting and diagnosing a case; this effort likely reflects active exploration of image areas toward identifying diagnostically relevant regions. In the future, monitoring tonic pupil diameter may also inform the timing and administration of medical decision support tools, interventions designed to aid interpretation and facilitate access to information and second opinions [25].

Phasic pupil diameter correlates with dynamic event-related variations in pupil diameter in response to viewing and exploiting information particularly relevant to interpretation. Though speculative, some theories suggest that phasic increases in pupil diameter may even be necessary for gathering the clinical information required for successful diagnosis [5, 6]. Data from this study provide some support this notion, demonstrating that pupil diameter is temporarily modulated by the difficulty of a case and reflects the pathologists’ diagnostic agreement with consensus reference diagnosis, possibly indicating the perceived diagnostic resolution of a case. Cases rated low difficulty tended to not elicit any positive- or negative-going deflection of pupil diameter upon arrival in a diagnostically relevant region of interest. Though entirely speculative, in relatively easy cases pathologists may have already arrived at successful interpretation prior to viewing a dROI. For instance, though benign and invasive cases may have particular regions most representative of those diagnoses, regions adjacent to the dROI likely hold similar (and easily extracted) informational value.

In contrast, cases rated high difficulty tended to elicit robust pupil diameter changes upon viewing a dROI. Interestingly, the positive- versus negative-going waveform reflected convergence versus divergence from the pathologists’ agreeing with the consensus reference diagnosis, respectively. When the pupil diameter waveform was positive-going upon viewing, pathologists ultimately delivered a diagnosis in agreement with consensus. It could be the case that this positive deflection reflects pathologists rendering particular regions of the image as highly relevant to their successful interpretation. Of course, this could occur prior to conscious awareness, affording the accurate perception and integration of relevant information [7]. In addition, we found a strong negative-going deflection when pathologists ultimately delivered a diagnosis different than the consensus reference diagnosis. This particular pattern was not hypothesized, though we believe it is compatible with some extant literature [6]: though speculative, when pathologists view a dROI but do not interpret it as relevant to their task, pupil diameter decreases and valuable visual details are not sufficiently processed. This failure to adequately identify regions as relevant to interpretation may ultimately result in assigning a less accurate diagnosis to the case. To our knowledge this is the first time such a pattern has been reported, extending current theories of pupil response [4, 5] and motivating continuing research into this phenomenon.

The present data suggest that phasic changes in pupil diameter may be used to monitor and guide the interpretive process. Using standardized cases with pre-determined regions of diagnostic relevance and consensus reference diagnoses, future computerized training platforms could monitor phasic pupil response and adaptively customize real-time feedback and cueing. For instance, if a trainee’s pupil diameter remains constant or shows a negative-going deflection upon fixation in a pre-determined dROI, they may have failed to identify a region as relevant to interpretation. Adaptive learning systems might leverage this information for more personalized, timely, and effective feedback [26].

Some limitations of the present study are worth considering. First, though our sample size is substantially larger than other eye tracking studies [1, 3, 27–29] using samples of medical practitioners, a sample with even greater breadth of pathology experience and specialization may reveal additional or different patterns of interest, allowing also an assessment of reliability for the present results. Second, though the present results were seen with breast biopsy images, we cannot draw any conclusions regarding whether the patterns of pupil size variation may generalize to other biopsy types or medical specialties (e.g., radiology). Third, while we adjusted for image brightness and encouraged a consistent seating distance from the monitor, future research might benefit from stabilizing the head with a chin rest. Indeed anterior-posterior head movement toward and away from the monitor during image interpretation might influence recorded pupil diameter. However, we note that the experimenter ensured a consistent participant seating position (60 cm from monitor) to maintain eye tracking quality, and remote eye trackers have been validated as reliable instruments for monitoring task-evoked pupil responses [30]. Fourth, future research may benefit from comparing pupil diameter responses elicited when pathologists view regions of interest established through consensus versus participant-specific regions of interest deemed of diagnostic relevance during intepretation [31]. Fifth, all data analyses were conducted and presented in aggregate format, without direct consideration of intra- and inter-individual differences in pupil response; to ensure applicability to individual readers, continuing research may benefit from investigating the reliability of the present patterns within individuals. Finally, though digital images (unlike glass microscopy) provide a tractable mechanism for eye tracking and are increasingly used for gathering second opinions [32], the U.S. Food and Drug Administration (FDA) has not yet approved digital whole slide images for the rendering of primary diagnoses.

In conclusion, we provide preliminary evidence that pupil diameter may prove valuable in monitoring pathologists’ interpretive process and reflecting agreement with consensus diagnosis during image interpretation. This result was found with tonic differences during the ongoing interpretive process, and more specifically with phasic differences in response to viewing diagnostically relevant image regions. These findings with physicians support theories of pupil response suggesting dynamic interactions between LC-NE function and the engagement of exploit-versus-explore cognitive control states. Uniquely, the present findings extend predictions made by these theories to the challenging real-world setting of medical decision making with high-stakes outcomes.

## Abbreviations

ANOVA, analysis of variance; DCIS, ductal carcinoma in situ; dROI, diagnostic region of interest; LC, locus-coeruleus; LCD, liquid crystal display; LC-NE, locus-coeruleus norepinephrine; FDA, Food & Drug Administration; RED-m, remote eye tracking device-mobile; TIFF, tagged image file format
